# Death of Sir Henry Halford

**Published:** 1844-05-18

**Authors:** 


DEATH OF SIR HENRY HALFORD.

At the mature age of 78, and capable of exercising his profession, with vigour, till a year ago, died Sir Henry Halford, the most eminent Physician in England—if the most extensive practice among the highest ranks in life be any criterion of evidence. Contemporary with Baille, the two stars of the medical horizon shone with nearly equal lustre; but with different kinds of light. Dr. Baille was sought for his real knowledge of MORBID ANATOMY, and his supposed knowledge of pathology, two things as different as cause and effect. His great competitor preferred no claim to deep science in either anatomy, physiology, or pathology; but he had a quick apprehension, an elegant address, a ready explanation for every phenomenon, a facile prescription for every symptom —in one word, he had TACT, of a very superior kind, which counterbalanced, and more than counterbalanced, all the drudgery of the dead-house, through which Dr. Baille had waded.

Then again, his polished manners, his classical education, and his double refined powers of flattery, gave him infinite advantage over his plain, homely, and unpolished rival, among the Royalty and the Aristocracy of the Kingdom, male and female. Dr. Baille was summoned in dangerous cases, through fear rather than favour; but Sir Henry was generally there long before the disease came to that pass, and consequently had the cream of the jest, while the pathologist had merely the honour of taking precedence of the undertaker.

Sir Henry's career lasted half a century, while that of Baillie ran only about 25 years. His figure was slight and elastic, his countenance animated and cheering, his activity of mind and body remarkable, and he had the art of inspiring hope in the breast of the expiring sufferer.

Of late years, his advanced age, his separation from Court, the death of most of his old patients, and the pressure of a redundant doctorate, curtailed greatly the range of his practice; but it is probable that he realized more money by his profession than did any physician since the days of Hippocrates. Quiescat in pace!—*Med. Chir· Rev.*

